# Editorial: Combining mechanistic modeling with machine learning to study multiscale biological processes

**DOI:** 10.3389/fsysb.2024.1367549

**Published:** 2024-02-02

**Authors:** Shayn Peirce-Cottler, Yoram Vodovotz

**Affiliations:** ^1^ Biomedical Engineering, Charlottesville, VA, United States; ^2^ Department of Surgery, University of Pittsburgh, Pittsburgh, PA, United States

**Keywords:** artificial intelligence, mechanistic model, parameterization, agent-based model (ABM), multiscale (MS) modeling

Biological and physiological processes occur across a broad spatiotemporal range, with processes at one level of scale (e.g., gene expression inside single cells) affecting processes at other levels of scale (e.g., coordinated migration of endothelial cells during angiogenesis and tumor growth). Deducing the cause-and-effect relationships that link biological and physiological mechanisms across scales is a major challenge that both machine learning (ML) and mechanistic modeling approaches seek to address. Mechanistic models are particularly well-suited for simulating and/or computing how abstracted, intersecting biological processes give rise to changes over time. Data-driven/machine learning (ML) approaches, such as neural networks and clustering algorithms, on the other hand, integrate massive amounts of data to identify patterns, trends, and correlations in the data. Both methodologies can be used to generate novel insights and testable hypotheses, though the means for doing so differ depending on the modeling approach. Emerging computational strategies are combining mechanistic modeling and ML in ways that capitalize on their unique attributes and compensate for the deficiencies of the other. As discussed in this Research Topic, the resulting synergy created by merging these methods more comprehensively and efficiently leverages large-scale data sets to produce new insights about *what* biological processes connect across spatial and temporal scales and *how* they intersect to drive changes in cells, tissues, and organs.

Sivakumar et al. provide foundational context for the integration of mechanistic and ML models, focusing on a particular class of the former (namely, agent-based models [ABM]). The authors introduce and explain key concepts, strengths, and limitations of both classes of models, and particularly highlight applications to spatial modeling of biological processes. They note the difficulties inherent in assessing ML models and discuss multiple applications of ML in the context of ABM (e.g., defining and determining agent rules, parameter estimation/model calibration, and reducing the computational cost of ABM).

Erdem and Birtwistle present another use case for integrating ML and mechanistic modeling, this time in the context of mining ‘omics data to define causal interactions and then integrate these inferences into a mechanistic model. The MEMMAL (MEchanistic Modeling with MAchine Learning) framework is presented by the authors, and in the example system ML models are used to generate a mechanistic model of intracellular pathways involving interferon-gamma and programmed cell death ligand (PDL)-1 based on inferred interactions among active genes, inactive genes, mRNA, and protein (including modified proteins).

Frank focused on a more fundamental question regarding how circadian rhythms are regulated at the transcriptional level using stochastic differential equations of transcription factor protein dynamics and the associated mRNAs that produce them, combined with an artificial neural network (ANN) encoding the cellular input-output function for greater efficiency in searching the solution space. Perhaps not unexpectedly, this study finds that multiple such solutions are possible. Nonetheless, this article sheds novel biological insights and brings up important parallels to over-parameterized ANNs in other settings of deep learning.

Li et al. demonstrate how neural networks can be used to accelerate a 3-dimensional finite element model (FEM) of morphogen-driven patterning in the zebrafish embryo. Impressively, the FEM represents all stages of zebrafish embryonic development using advection-diffusion-reaction Partial Differential Equations (PDEs) in a growing domain. However, solving the PDEs is computationally expensive and time-consuming, so the authors developed an accurate and fast Neural Network (NN) surrogate model of the 3D embryo that enabled more rapid parameter exploration. This allowed them to investigate the multifaceted mechanisms of bone morphogenic protein (BMP)-mediated embryonic morphogenesis and highlighted, for the first time, the importance of advection in the formation of the BMP gradient that regulates morphogenic patterning.

King et al. introduce a novel optimization approach, termed “pathway—controlled optimization (PCO)”, to predict regulation of cell metabolism using a thermodynamic framework. The authors’ approach extends their prior published work, which introduced optimization and reinforcement learning methods to predict the enzymes that must be regulated in order to maintain metabolite concentrations within ranges that satisfy reaction-diffusion limits. One advantage of this approach is that relatively few experimental measurements are needed for parameter identification. The authors apply their method to the growth of *Rhodospirillum rubrum*, a photosynthetic bacterium which produces ethylene. By including cell growth as an objective for metabolism regulation, the method predicted that regulation favors higher rates of protein and RNA synthesis over DNA synthesis for most of the cell cycle, which is in agreement with experimental observations.

An and Cockrell contribute a novel hypothesis and theoretical framework for generating synthetic, multi-dimensional molecular time-series data for neural network-based, artificial intelligence (AI) representations of biomedical systems. The authors make the case that when it is necessary to know how the biomedical system works (e.g., when the goal is to understand biomarkers of disease and drug mechanisms), current statistical and ML approaches for generating synthetic data are insufficient. They suggest that complex, multiscale mechanism-based simulation models are useful for generating synthetic data that can minimize the typical limitations of neural network AI systems, such as overfitting and lack of generalizability. As a demonstration, the authors use a published cell-level agent-based model (ABM) within a ML-augmented pipeline to create synthetic cytokine trajectories in trauma patients that either do or do not develop acute respiratory distress syndrome. An important contribution is the enumeration of specific properties that mechanistic models must possess in order to be useful for generating synthetic molecular time series data for training neural networks.

A key high-level theme that emerges from this Research Topic is that combining mechanistic modeling with ML yields advantages in terms of compute time, parameterization, and data generation for AI representations of biomedical systems. Also evident in this Research Topic of papers is the broad diversity of contexts across which mechanistic modeling and ML have been combined to study complex and multiscale biological processes–from programmed cell death, to circadian rhythm regulation, to embryo morphogenesis, to bacterial metabolism, to acute respiratory distress syndrome in patients and that while work in this area is still nascent, continued exploration, development, and validation of novel approaches can yield new understanding of complex biological and biomedical processes.

